# Multi-omics integration and machine learning identify a novel gene signature consisting of CCDC141, CHI3L2, RIMKLB, and PDLIM7 in bronchopulmonary dysplasia associated with neutrophil-driven immune dysregulation

**DOI:** 10.1016/j.clinsp.2026.101059

**Published:** 2026-07-14

**Authors:** Yanchun Sun, Jingye You, Jinhao Tao, Jiale Dai, Aiju Chen, Fei Luo, Yun Chen

**Affiliations:** a Department of Pediatric Medicine, StarKids Children's Hospital, Shanghai, China; b Department of Neonatology, Obstetrics & Gynecology Hospital of Fudan University, Shanghai Key Lab of Reproduction and Development, Shanghai Key Lab of Female Reproductive Endocrine Related Diseases, Shanghai, China; c Pediatric Intensive Care Unit, Children's Hospital of Fudan University, Shanghai, China

**Keywords:** Bronchopulmonary dysplasia, Diagnostic biomarkers, Machine learning, Mendelian randomization, Neutrophils, Immune dysregulation, PDLIM7, Preterm infant, Bioinformatics

## Abstract

**Background:**

Bronchopulmonary Dysplasia (BPD) is a major complication in preterm infants, lacking effective diagnostic biomarkers to facilitate early detection and intervention.

**Methods:**

We employed a multi-omics approach to identify diagnostic biomarkers for BPD. Analysis of the GEO dataset GSE32472 revealed 273 Differentially Expressed Genes (DEGs). Weighted Gene Co-expression Network Analysis (WGCNA) identified co-expression modules. A diagnostic model was developed using a machine learning pipeline that sequentially integrated LASSO regression, Support Vector Machine (SVM), and random forest algorithms. Feature selection through machine learning identified a four-gene diagnostic signature. We conducted Gene Set Enrichment Analysis (GSEA) and immune cell infiltration assessment via CIBERSORT. Additionally, a two-sample Mendelian Randomization (MR) analysis explored the potential causal relationship between serum neutrophil counts and BPD-related lung impairment. Experimental validation employed a BPD animal model with H&E staining, TUNEL assay, RT-qPCR, and Western blotting.

**Results:**

WGCNA identified 21 co-expression modules, with the red module showing the strongest correlation with BPD. Machine learning feature selection identified a four-gene diagnostic signature: CCDC141, CHI3L2, PDLIM7, and RIMKLB. This signature demonstrated significant diagnostic potential (AUC > 0.8), validated in an external dataset. GSEA indicated associations with immune and metabolic pathways. Furthermore, MR analysis suggested a causal link between higher serum neutrophil counts and reduced risk of BPD-related lung impairment.

**Conclusions:**

CCDC141, CHI3L2, PDLIM7, and RIMKLB are identified as promising diagnostic biomarkers for BPD ‒ potentially involved in immune-related pathogenesis.

## Highlights

• A novel 4 ‒ gene signature (CCDC141, CHI3L2, PDLIM7, RIMKLB) is identified for Bronchopulmonary Dysplasia.
• The signature shows high diagnostic accuracy (AUC > 0.8) and links to immune cell infiltration.
• Mendelian randomization proves causal effect of neutrophil count on lung function.
• Integration of computational genomics and experimental validation reveals immune mechanisms.

## Introduction

Bronchopulmonary Dysplasia (BPD), a chronic lung disease affecting 35% of preterm infants annually, is predominantly observed in very low-birth-weight infants after supplemental oxygen therapy and mechanical ventilation.^1^^,^^2^ The pathogenesis of BPD is multifaceted, with inflammation playing a central role.^3^ BPD pathology is characterized by decreased alveolar and capillary counts, structural simplification and enlargement of alveoli, and thickening of the basement membrane. Alveolar and pulmonary microvascular hypoplasia are key pathological features of BPD and remain a current focus of research.^4^ Among the numerous implications of BPD, enduring lung function impairment, which may persist into adulthood, and compromised neurodevelopment are of greatest concern.^5^ Currently, there is an absence of specific preventive or therapeutic agents for BPD.^6^ Therefore, it is critical to explore in depth the pathogenesis and treatment strategies of BPD.

Although initially derived from artificial intelligence research, machine learning models have progressively established a significant role in the analysis of vast volumes of intricate biological data in recent years.^7^ Consequently, they have found successful applications in disease diagnosis, drug screening, and basic research.^8^ In the domain of protein function, the introduction of machine learning into analytical models has enhanced prediction precision and deepened functional analysis.^9^ Similarly, in metabolic engineering, machine learning has refined data analysis techniques, thereby saving time and augmenting the accuracy of predictions of metabolic outcomes.^10^ Tools such as Random Forest (RF) have been utilized to assess variable importance and model predictions. Likewise, Least Absolute Shrinkage and Selection Operator (LASSO) has found extensive use in association mapping studies.^11^^,^^12^ Support Vector Machine (SVM) approaches have been used extensively in pattern recognition and classification.^13^ The application of these machine learning models to disease target gene analysis and the prediction of characteristic genes associated with diseases represents a novel approach.

This study used multiple bioinformatics methods to identify characteristic genes in BPD. These genes showed good diagnostic performance and were validated by external data sets. The signaling pathways enriched by these genes and their role in immune cell infiltration were further evaluated. Finally, Real-Time quantitative PCR (RT-qPCR), and Western blot were used to verify the expression of hub genes in BPD animal models. This verification provides new insights into the role of key genes in the pathogenesis of BPD and supports subsequent targeted intervention studies.

## Materials and methods

### Data sources

This study utilized two distinct datasets for comprehensive analysis: GSE32472 (Platform: GPL6244, [HuGene-1_0-st] Affymetrix Human Gene 1.0 ST Array [transcript (gene) version]) and GSE108754 (Platform: GPL13497, Agilent-026652 Whole Human Genome Microarray 4 × 44 K v2 [Probe Name version]). These datasets were sourced from the Gene Expression Omnibus (GEO), available at http://www.ncbi.nlm.nih.gov/geo/. The GSE32472 dataset, which was used as the training set, includes microarray-based gene expression evaluations approximately on days-5, −14, and −28 of life. To ensure diagnostic consistency, a definitive BPD diagnosis was established based on 100 blood samples collected around day-28 of life. These samples were derived from 38 control subjects, 38 mild BPD patients, 10 moderate BPD patients, and 14 severe BPD patients. For external validation, the GSE108754 dataset was used, which comprises five neonates diagnosed with BPD and six healthy controls.

### Data preprocessing and batch effect correction

To address batch effects and platform-specific biases, we used the ComBat tool from the Sangerbox platform (http://sangerbox.com/). The dataset was first uploaded. Batch effect correction was then applied to harmonize variations across different platforms (Affymetrix, Agilent, and Illumina) and sample types (blood, umbilical cord blood). Data were then normalized for comparability, and quality checks confirmed that batch effects were adequately corrected.

### Identification of DEGs

The identification of DEGs was conducted using the ‘limma’ package in R software.^14^ The criterion for this analysis was an adjusted p-value < 0.05 and an absolute log Fold Change (logFC, base 2) > 0.585. A volcano plot was generated to visualize these DEGs. Furthermore, the top 50 up-regulated and the top 50 down-regulated DEGs were displayed in a heatmap.

### Functional and pathway enrichment analyses

The clusterProfiler package in R was utilized for the functional enrichment analyses of DEGs using Gene Ontology (GO) and Kyoto Encyclopedia of Genes and Genomes (KEGG). The GO analysis identified three categories: Biological Processes (BP), Cellular Components (CC), and Molecular Functions (MF). These categories facilitated the exploration of biological functions associated with the DEGs, while potential signaling pathways related to the DEGs were investigated through KEGG analysis.

### WGCNA

The co-expression network in the GSE32472 cohort was constructed using WGCNA, adhering to the scale-free topology criterion. The pickSoftThreshold function of the WGCNA package was utilized to calculate the soft-thresholding power (β) and adjacency matrix. Following this, the adjacency matrix was converted into a Topological Overlap Matrix (TOM), and the corresponding dissimilarity was calculated to perform hierarchical clustering analysis. The dynamic tree cutting method was used to identify co-expressed gene modules, with a minimum module size of 50. Module Eigengenes (MEs), which are the principal components representing gene modules, were calculated to summarize the expression profiles of genes within each module. The correlations between module eigengenes and immune cell types were computed to identify relevant gene modules. Modules that were highly correlated with a given immune cell were selected for further analysis. Soft-thresholding power (β) was selected as the lowest value achieving scale-free topology fit R^2^ > 0.8, following WGCNA standards.

### Identification of signature genes

We pinpointed potential core genes via the intersection of DEGs and essential module genes. Subsequently, we applied three machine learning algorithms ‒ specifically LASSO, Random Forest, and Support Vector Machine (SVM) ‒ for core gene screening.

The LASSO analysis was conducted using the glmnet package, with penalty parameters tuned using 10-fold cross-validation. This method is superior to traditional regression analysis when evaluating high-dimensional data. In addition, we utilized the R package “randomForest” to classify the DEGs for hub genes. The Random Forest model determined the optimal number of variables by calculating the average error rate of the candidate hub genes. Subsequently, we computed the error rate for each number of trees from 1 to 500 and determined the optimal number of trees based on the tree with the lowest error rate. A Random Forest model was constructed once these parameters were established. Ultimately, the feature importance scores of each candidate hub gene were identified, and genes with an importance value (mean decrease Gini) exceeding 0.25 were selected.

SVM has drawn wide attention over the last two decades due to its extensive applications and has shown highly advantageous performance in various tasks. SVM is designed based on the principle of structural risk minimization, with the central concept being to establish a maximum margin between two classes of data. We used the SVM function of the e1071 R package with default parameters. The final prediction score was obtained by averaging the outputs from multiple SVM models trained during cross-validation.

The intersecting genes identified by all three machine learning algorithms were considered the signature genes of children with Bronchopulmonary Dysplasia (BPD). The area under the Receiver Operating Characteristic (ROC) Curve (AUC) was utilized to evaluate the diagnostic efficiency of these signature genes. An AUC over 0.7 indicated favorable diagnostic performance.

### Gene set enrichment analysis (GSEA)

To discern the relationship between signature genes and signaling pathways, we classified the Bronchopulmonary Dysplasia (BPD) cohort based on the median expression of the hub genes. We then conducted GSEA on the resulting subgroups, using an adjusted p-value cutoff between 0.05 and 0.1.

### Immune cell infiltration

In this study, we employed the Cell-type Identification By Estimating Relative Subsets Of RNA Transcripts (CIBERSORT) method, which uses the principle of ν-support vector regression to deconvolute the expression matrix of 21 human immune cell subtypes. This approach was used to determine differences in immune cell infiltration between children diagnosed with bronchopulmonary dysplasia (BPD) and healthy individuals. Subsequently, we identified the immune cells that exhibited significant differences in infiltration between neonates diagnosed with bronchopulmonary dysplasia (BPD) and matched controls. The correlation between these immune cells and the previously identified signature genes was then evaluated using Spearman's rank correlation coefficient.

### Mendelian randomization (MR)

Genetic instrument selection and Mendelian randomization analysis were conducted using publicly available GWAS summary statistics from open databases. Instrumental Variables (IVs) comprise Single Nucleotide Polymorphisms (SNPs) significantly associated with hub genes (p < 5 × 10–8). Data harmonization and analysis were performed via the TwoSampleMR R package (v0.5.7). To ensure IV independence, Linkage Disequilibrium (LD) clumping was applied with default parameters (“clump = TRUE”; r2 < 0.001 within 10,000 kb genomic windows). SNPs with F-statistic < 10 were excluded to mitigate weak instrument bias. Two-sample MR was employed to assess causal relationships between hub genes (e.g., neutrophil-related markers) and lung function (FEV1/FVC). The primary inference was based on the Inverse-Variance Weighted (IVW) method.

Sensitivity analyses included the following methods: 1) MR-Egger regression to test directional pleiotropy (intercept p < 0.05 indicating significance); 2) MR-PRESSO Global Test to detect horizontal pleiotropy via Residual Sum of Squares (RSS) deviation (p < 0.05), with iterative removal of outlier SNPs (outlier p < 0.05) followed by re-running IVW; 3) Cochran’s *Q* statistic for heterogeneity assessment (p < 0.05 indicating heterogeneity); 4) Leave-one-out analysis to verify estimate stability after excluding individual SNPs.

MR-PRESSO was further employed for comprehensive outlier detection and horizontal pleiotropy correction, enhancing the robustness of causal inferences. All retained SNPs satisfied F-statistic > 10, confirming robust instrument validity.

### Animals and BPD model establishment

Female (20–25 g) and male (25–30 g) C57BL/6 mice, aged 2–3 months, were sourced from Chengdu Duosai Experimental Animal Co., Ltd. (Sichuan). Prior to experimentation, mice were habituated for one week in standard cages with unrestricted access to food and water. Mice were randomly allocated to cages, each containing males and females at a 2:1 ratio. Housing conditions were maintained at 22±2 °C, 50%–60% relative humidity, with a 12-hour light-dark cycle. Furthermore, full-gestation C57BL/6 pups at embryonic days-20 to −21 (E20–E21), near term but still in utero, were included in the study. Mice were randomly assigned to experimental groups. Radio-Frequency Identification (RFID) microchips were used throughout experimentation and data interpretation to keep researchers blinded to treatment allocations. This study was conducted in full compliance with both the National Standard of China (GB/T 35892-2018) and the international ARRIVE 2.0 guidelines. The animal procedures received prior approval from the Animal Ethics Committee of Fudan University (Approval n° 2023-213), and detailed documentation is available in the Supplementary Materials.

### Hematoxylin-eosin (H&E) staining

The lung tissue was fixed with 4% formaldehyde at ambient temperature for 48 h, then dehydrated in a graded alcohol series, with each step lasting 2-min. Following xylene-mediated clarification, the tissue was embedded in paraffin. The paraffin blocks were then cut into 4 µm sections, which underwent xylene dewaxing twice, rehydration through a graded ethanol series, and immersion in distilled water for 5-min. Hematoxylin staining was applied for 10 min, followed by a rinse in distilled water for 1 hour. The sections were then dehydrated again in a graded alcohol series for 10 min and counterstained with 1% eosin-alcohol solution for 3–5 min. After staining, the sections were dehydrated with absolute ethanol, cleared with xylene, and finally mounted using a resinous mounting medium. Microscopic examination and photography were performed to observe the tissue morphology.

### Terminal deoxynucleotidyl transferase dUTP nick end labeling (TUNEL) assay

Previously established methods were employed to prepare paraffin sections. The sections underwent a permeabilization step involving the addition of 0.1% Triton X-100 (Solarbio, China) for 5-min. Following this, a 3% H_2_O_2_ solution was prepared and applied to block endogenous peroxidase activity. The sections were washed three times in PBS at 25±2 °C, each wash lasting 10-min. Apoptosis detection was performed using the TUNEL assay with detection and core kits from KeyGEN (Jiangsu, China). Color development was achieved using a DAB working solution as the chromogen in immunohistochemistry; this step was followed by three consecutive PBS washes. Counterstaining was performed with either hematoxylin or methyl green, and the sections were mounted with a neutral mounting medium. Additionally, for co-labeling experiments, the Servicebio CF488 TUNEL apoptosis detection kit was utilized.

### Real-time polymerase chain reaction (RT-qPCR) assay

Total RNA was extracted from lung tissues using a total RNA extraction kit (Beijing Solabo Technology Co., Ltd., Cat. n° R1200) according to the manufacturer's instructions. First-strand complementary DNA (cDNA) was synthesized from 1 µg of total RNA using the ImProm-II™ Reverse Transcription System (Promega Corporation). qPCR reactions were performed on an ABI PRISM 7000 Sequence Detection System (Shanghai Pudi Biotechnology Co., Ltd.) using SYBR Green Real-time PCR Master Mix (Toyobo Life Sciences). PCR thermal cycling conditions were as follows: an initial denaturation step at 95 °C for 60 s; followed by 40 cycles of 95 °C for 15 s, annealing at 60 °C for 15 s, and an extension step at 72 °C for 45 s. GAPDH was used as an internal control. Relative messenger RNA (mRNA) expression of target genes was determined using the 2^^-ΔΔCq^ method.

### Western blot analysis

The lung tissue specimens underwent a two-stage cryopreservation process. They were initially stored in a −20 °C freezer for 6-hours, followed by long-term storage in a −80 °C freezer until further experimentation. Protein extraction from these tissues was accomplished by employing a commercially available RIPA lysis buffer sourced from Elabscience Biotechnology Inc. Protein concentrations were then measured using the Bicinchoninic Acid (BCA) assay supplied by Beijing Solabio Technology Co., Ltd. After electrophoretic separation on a 15% SDS-PAGE gel, 30 µg of protein was transferred onto PVDF membranes, which were subsequently blocked with 5% skim milk for one hour. Target protein detection was performed by incubating the blocked membranes overnight at 4 °C with a primary antibody specific to glyceraldehyde 3-phosphate dehydrogenase (GAPDH; dilution 1:5000, ab201822, Abcam). After thorough washing with TBS-T buffer (0.1% Tween 20) three times for 10-minutes each, the membranes were incubated for 1-hour with a horseradish peroxidase-conjugated goat anti-rabbit IgG secondary antibody (dilution 1:2000, ab6721, Abcam). GAPDH served as an internal loading control. Protein bands were detected using an enhanced ECL chemiluminescent substrate kit from Shanghai Yesen Biotechnology Co., Ltd. (China), and the resulting images were analyzed with the ChemiDoc imaging system manufactured by Bio-Rad Laboratories Inc.

### Statistical analysis

**Fig. 1:**
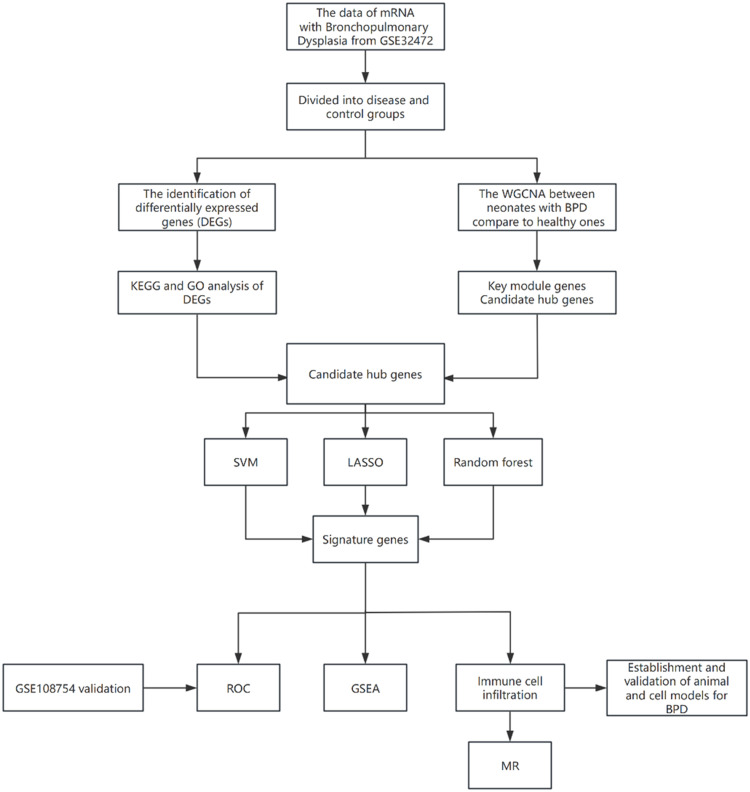
The flow chart of this research.

All statistical evaluations in this study were performed using R software (version 4.3.2). Unless otherwise specified, a p-value of <0.05 was considered statistically significant, and all p-values were calculated using two-tailed tests. The research flowchart is shown in Fig. 1.

## Results

### Expression of CRGs in BPD

**Fig. 2:**
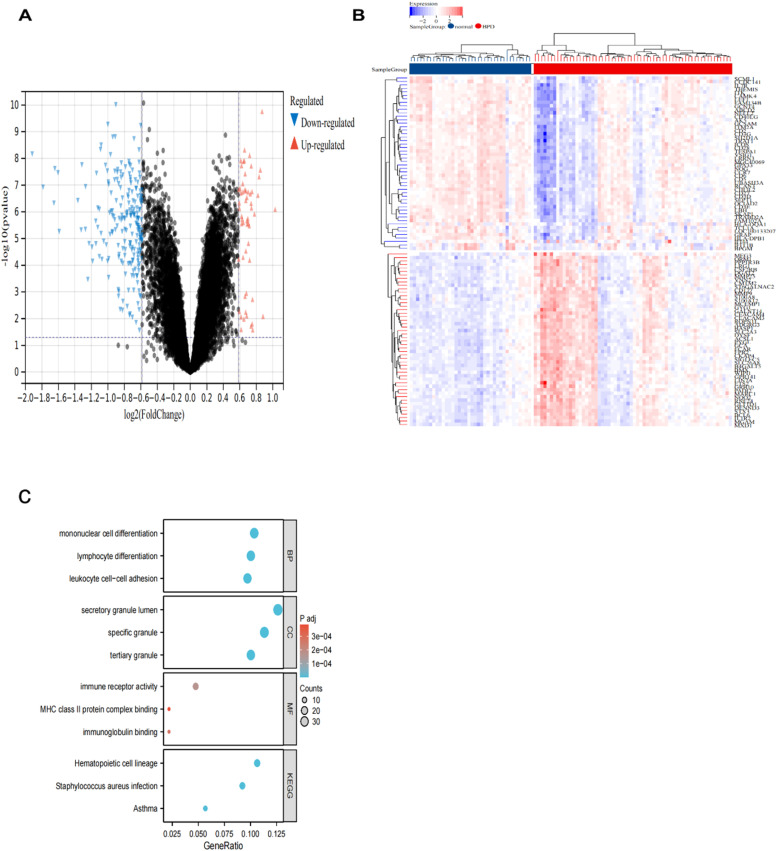
Identification of the differentially expressed genes (DEGs) in BPD. (A) Volcano showed expression of DEGs between BPD and healthy. (B) The heatmap showed the DEGs. (C) Functional enrichment anatysis of DEGs. The top 3 functional enrichment in Biological Process (BP), Cellular Component (CC), and Molecular Function (MF) analysis and Kyoto Encyclopedia of Genes and Genomes (KEGG) analysis of DEGs.

We analyzed DEGs from neonates with BPD and healthy controls using the “limma” package. A total of 273 DEGs were ultimately identified, with 58 genes up-regulated and 215 genes down-regulated (Fig. 2A). The heatmap displays the top 50 DEGs with the greatest up-regulation and the top 50 with the greatest down-regulation in BPD neonates compared to healthy controls (Fig. 2B).

### Enrichment analysis of functions

The Gene Ontology (GO) analysis is categorized into three sections: Biological Process (BP), Cellular Component (CC), and Molecular Function (MF), as shown in Fig. 2C. The BP analysis reveals significant enrichment in processes such as positive regulation of mononuclear cell differentiation, lymphocyte differentiation, and leukocyte cell-cell adhesion. In the CC category, the top three terms are the secretory granule lumen, specific granule, and tertiary granule. In the MF category, immune receptor activity, MHC class II protein complex binding, and immunoglobulin binding are identified as key functions. Additionally, the Kyoto Encyclopedia of Genes and Genomes (KEGG) analysis highlights the top three enriched pathways: transcriptional misregulation during Staphylococcus aureus infection, hematopoietic cell lineage, and asthma, as depicted in Fig. 2C.

### Construction of the weighted gene co-expression network

**Fig. 3:**
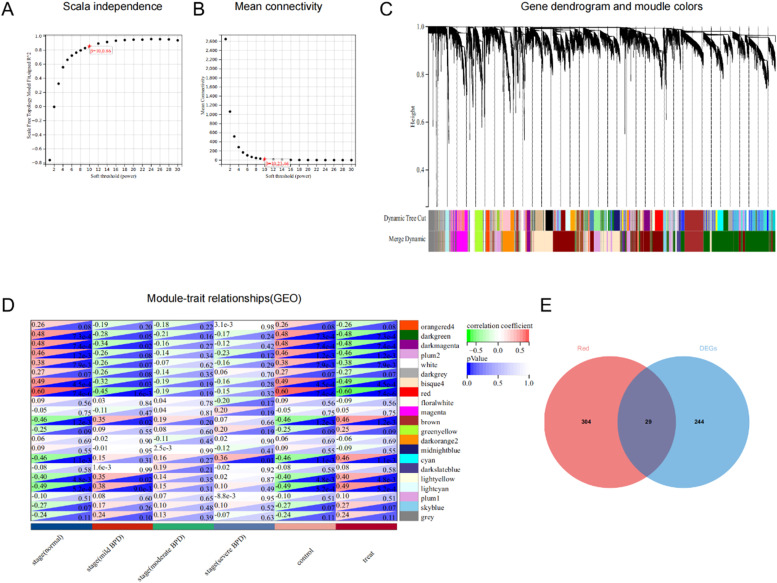
The weighted gene co-expression network analysis (WGCNA) analysis of GSE32472 and identification of candidate hub genes. (A) The soft threshold power of WGCNA. (B) The mean connectivity of WGCNA. (C) The cluster dendrogram of WGCNA. (D) The clustered modules of WGCNA. (E) The veen plot showed the interaction between DEGs and genes in red module.

Neonates diagnosed with BPD and healthy control neonates were subjected to analysis utilizing the WGCNA package within the R software. Following this, a scale-free co-expression network was constructed with the soft-threshold power determined as 16. This network exhibited a scale-free index of 0.86 and a reasonably satisfactory mean connectivity (Fig. 3A‒B). The cluster dendrogram (Fig. 3C) was used to segment the data into 21 modules (Fig. 3D). The correlation between each module and the neonates diagnosed with BPD group was then calculated. It was found that the red module exhibited a significant correlation with neonates diagnosed with BPD (correlation = −0.60, p < 0.0001). The red module, encompassing 333 genes, was therefore deemed a key module significantly associated with BPD in neonates. The overlap between the Differentially Expressed Genes (DEGs), and genes within the red module is exhibited in Fig. 3E.

### Utilizing LASSO, SVM and random forest algorithms for signature gene selection

**Fig. 4:**
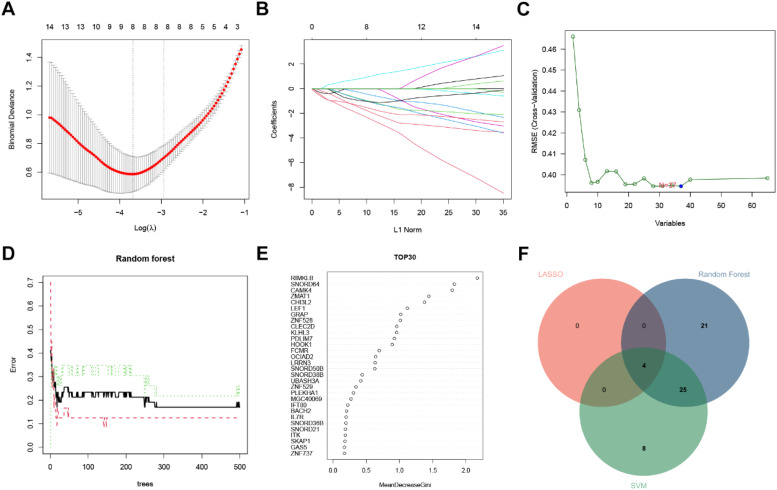
The machine algorithms for signature genes. (A) Penalty plot of the Least Absolute Shrinkage and Selection Operator (LASSO) model with error bars denoting standard errors. (B) LASSO plot showed the variations in the size of coefficients for parameters shrank as the value of k penalty increased. (C) Support Vector Machine (SVM’s) RMSE during cross-validation as the x-axis numbers change. (D) The error rate confidence intervals for random forest model. (E) The relative importance of genes is >0.25 in random forest model. (F) The interaction of the LASSO, SVM and random forest algorithms.

In this research, we utilized the LASSO machine learning algorithm to identify unique genes associated with BPD diagnosis in newborns from the previously identified significant candidate genes. Meanwhile, we applied the SVM and Random Forest machine learning methods to select feature genes based on distinguishing between disease and control groups. Subsequently, we determined the intersection of the genes selected by the three techniques to identify common feature genes. As depicted in Figs. 4A and B, the LASSO algorithm identified eight feature genes. Conversely, the Random Forest algorithm identified 47 feature genes with relative importance exceeding 0.1. However, only the top 30 genes are displayed (refer to Figs. 4D and E). During SVM training, the default Radial Basis Function (RBF) kernel was employed, which yielded the most effective results by identifying 37 feature genes (refer to Fig. 4C). By combining the results of the three algorithms, we ultimately identified four feature genes: CCDC141, CHI3L2, PDLIM7, and RIMKLB (Fig. 4F).

### Diagnostic efficacy of signature genes in BPD

**Fig. 5:**
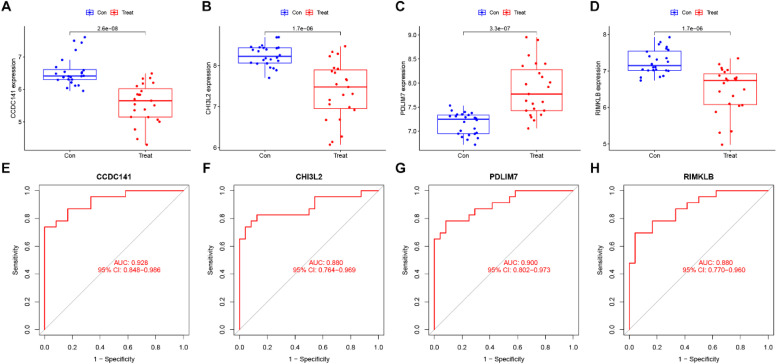
The performance of the signature genes in GSE32474. (A‒D) The expression of signature genes between the Neonatal BPD and healthy cohort. (E‒H) Receiver Operating Characteristic (ROC) showed the diagnostic performance of the signature genes.

**Fig. 6:**
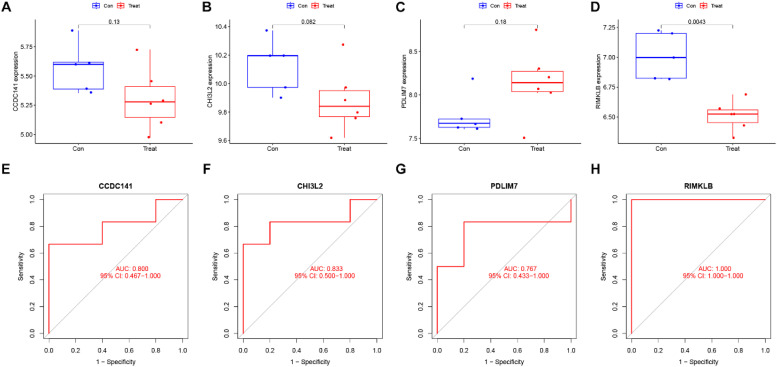
The performance of the signature genes in GSE108754. (A‒D) The expression of signature genes between the Neonatal Bronchopulmonary Dysplasia (BPD) and healthy cohort. (E‒H) ROC showed the diagnostic performance of the signature genes.

The genes we identified in our study were found to be significantly overexpressed in neonates diagnosed with BPD, compared to their healthy counterparts, suggesting a potential role of these genes in the pathogenesis of the disease (Figs. 5A‒D). Moreover, the Area Under the ROC Curve (AUC) for these genes was 0.928 for CCDC141, 0.880 for CHI3L2, 0.900 for PDLIM7, and 0.880 for RIMKLB, respectively (Fig. 5E‒H). To further assess the diagnostic efficiency of each gene, we analyzed an external validation cohort. Consistent with the GSE108754 dataset, these genes were markedly overexpressed in neonates diagnosed with BPD (Fig. 6A‒D). The AUC values in the validation set were 0.800 for CCDC141, 0.833 for CHI3L2, 0.767 for PDLIM7, and notably, RIMKLB achieved a perfect AUC of 1.00. In conclusion, the genes identified in our study could potentially be linked to the development and progression of BPD.

### GSEA

**Fig. 7:**
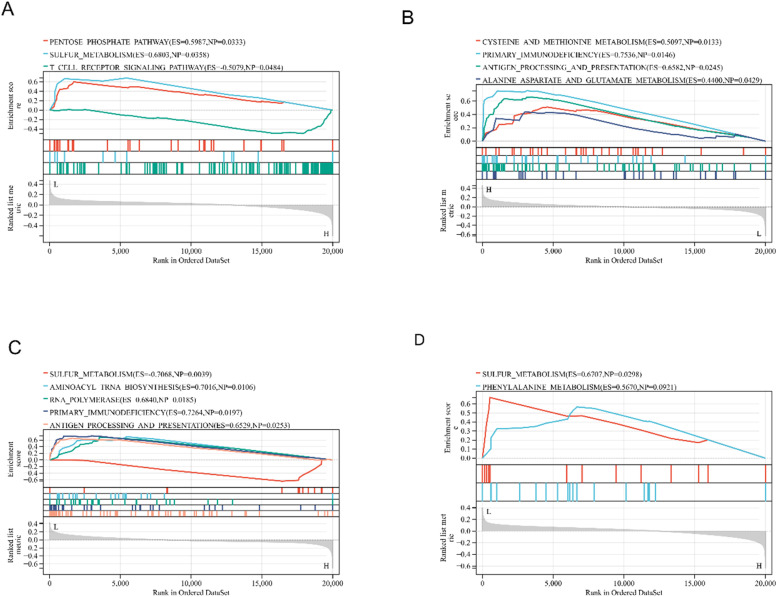
The gene set enrichment analysis (GSEA) of the signature genes in Neonatal BPD. (A) The GSEA of CCDC141 in Neonatal BPD. (B) The GSEA of CHI3L2 in Neonatal BPD. (C) The GSEA of PDLIM7 in Neonatal BPD. (D) The GSEA of RIMKLB in Neonatal BPD.

We conducted an evaluation of signaling pathways associated with signature genes via GSEA. The top pathways are presented in Fig. 7. CCDC141 is significantly associated with the T-cell receptor signaling pathway, sulfur metabolism, and the pentose phosphate pathway (Fig. 7A). CHI3L2 expression is significantly associated with antigen processing and presentation, primary immunodeficiency, alanine, aspartate and glutamate metabolism, cysteine and methionine metabolism (Fig. 7B). Furthermore, PDLIM7 expression is significantly associated with aminoacyl-tRNA biosynthesis, antigen processing and presentation, primary immunodeficiency, sulfur metabolism, and RNA polymerase (Fig. 7C). Moreover, RIMKLB expression is significantly associated with phenylalanine metabolism and sulfur metabolism (Fig. 7D). In summary, sulfur metabolism emerged as the most prominent shared pathway, significantly associated with three of the four genes: CCDC141, PDLIM7, and RIMKLB; meanwhile, antigen processing and presentation and primary immunodeficiency were both significantly enriched for two genes: CHI3L2 and PDLIM7.

### Immune cell infiltration

**Fig. 8:**
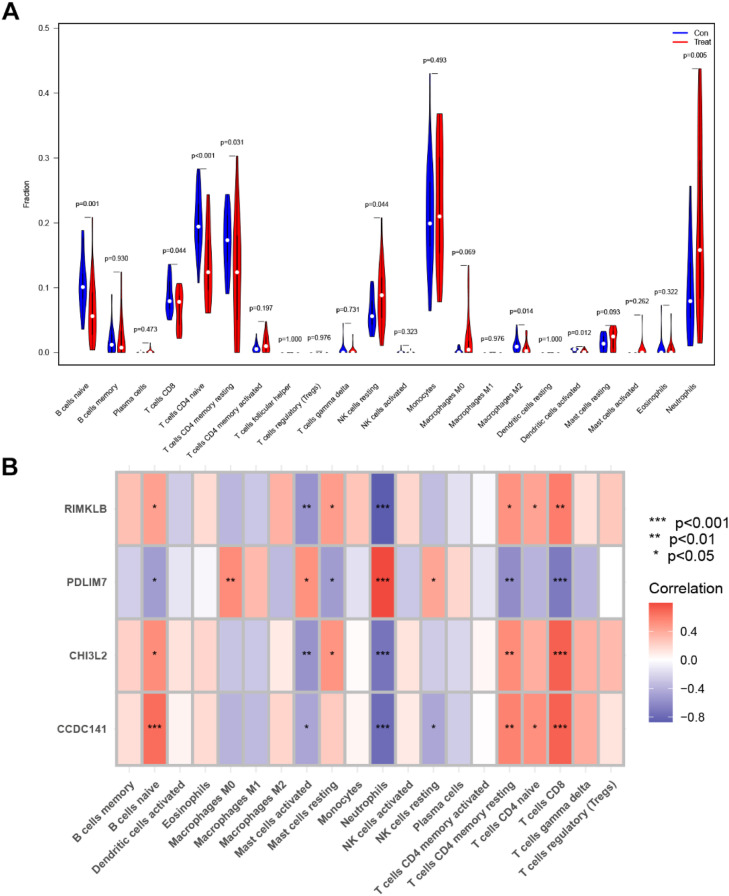
The immune cell infiltration association with signature genes. (A) The immune cell infiltration between the Neonatal BPD and healthy cohort. (B) The association between signature genes and significantly different immune cell infiltration. “ns” means (p ≥ 0.01). *p < 0.01, **p < 0.001, and ***p < 0.0001.

The immunological characteristics were assessed based on the extent of immune cell infiltration. Neonates diagnosed with BPD exhibited a heightened infiltration of neutrophils and resting memory CD4 T-cells compared to their healthy counterparts. Conversely, diminished levels of naive B cells, CD8 T-cells, naive CD4 T-cells, resting memory CD4 T-cells, M2 macrophages, and activated dendritic cells were observed (Fig. 8A). Correlation analysis revealed that the expressions of CCDC141, CHI3L2, and RIMKLB were positively correlated with CD8 T-cells, resting memory CD4 T-cells, resting mast cells, and naive B-cells, while PDLIM7 showed a negative correlation with these cell types. In contrast, CCDC141, CHI3L2, and RIMKLB were negatively correlated with neutrophils, activated mast cells, M0 macrophages, and resting NK cells, whereas PDLIM7 exhibited a positive correlation with these cells (Fig. 8B).

### The causal relationship between neutrophils and the risk of lung function (FEV1/FVC) was investigated

**Fig. 9:**
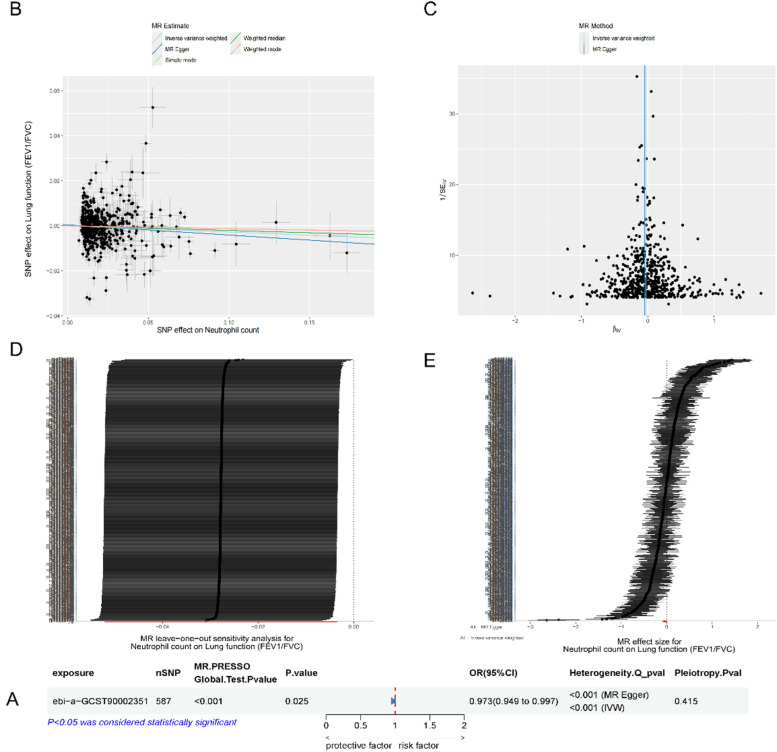
MR study results. (A) Forest plot showing the main causal effect estimate obtained using the Inverse-Variance-Weighted (IVW) method. Genetically predicted higher neutrophil count (ebi-a-GCST90002351) was associated with a reduced risk of impaired lung function (FEV1/FVC) (OR = 0.973, 95% CI 0.949–0.997, p = 0.025). (B) Scatter plot of polymorphism(SNP) effects on the exposure (neutrophil count) against effects on the outcome (lung function). Lines represent fits from different MR methods (IVW, MR-Egger, weighted median, etc.). X-axis is “SNP effect on Neutrophil count” and the Y-axis is “SNP effect on Lung function (FEV1/FVC)”. (C) Funnel plot to assess heterogeneity and potential directional pleiotropy among the instrumental variable SNPs. (D) Leave-one-out sensitivity analysis plot showing the stability of the causal estimate when sequentially removing each SNP. (E) Cumulative forest plot displaying the consistent directional effect of all individual SNPs on the protective association after outlier removal.

Primary Mendelian randomization analysis using the Inverse-Variance Weighted (IVW) method (Fig. 9A) demonstrated that genetic predisposition to neutrophils (ebi-a-GCST90002351) significantly reduced disease risk (OR = 0.973, 95% CI 0.949–0.997, p = 0.025), supported by 587 instrumental SNPs (all F-statistics > 10). Scatter plot visualization (Fig. 9B) confirmed concordant inverse associations across IVW, MR-Egger, and weighted median methods, with parallel IVW and MR-Egger trajectories (P_intercept = 0.415) excluding directional pleiotropy despite heterogeneity-driven dispersion at high exposure magnitudes (X > 0.1). Funnel plot asymmetry (Fig. 9C) revealed a left-skewed SNP distribution, characterized by dense clustering at β_IV 〈 2 and 1/SE_IV < 5, and sparse dispersion at β_IV 〉 5, indicating biological heterogeneity (IVW Q p-value < 0.001) rather than publication bias. The MR-PRESSO global test (p < 0.001) confirmed robustness after outlier removal by correcting for influential SNPs. Leave-one-out analysis (Fig. 9D) demonstrated exceptional stability, with resistance to single-SNP exclusion validated by MR-PRESSO (OR range: 0.970–0.978; maximum change in OR = 0.005; all 95% CIs < 1.0). The cumulative forest plot (Fig. 9E) corroborated cumulative protection: all 587 SNPs showed consistent positive effect sizes (β > 0; no null-line crossing) after MR-PRESSO outlier removal, plateauing at β = +0.80 (95% CI 0.72–0.88, p < 1 × 10^−10) with narrow confidence intervals (maximum width < 1.2 units). The convergence of IVW estimates, sensitivity analyses, and visualization plots substantiated the causal protective role of neutrophils (ebi-a-GCST90002351). MR-PRESSO resolved heterogeneity-driven asymmetry (p < 0.001), while the absence of pleiotropy (p = 0.415) and leave-one-out stability (ΔOR < 0.005) fulfilled STROBE-MR criteria for robust causal inference.

### Verification of key marker expression in BPD model mice

**Fig. 10:**
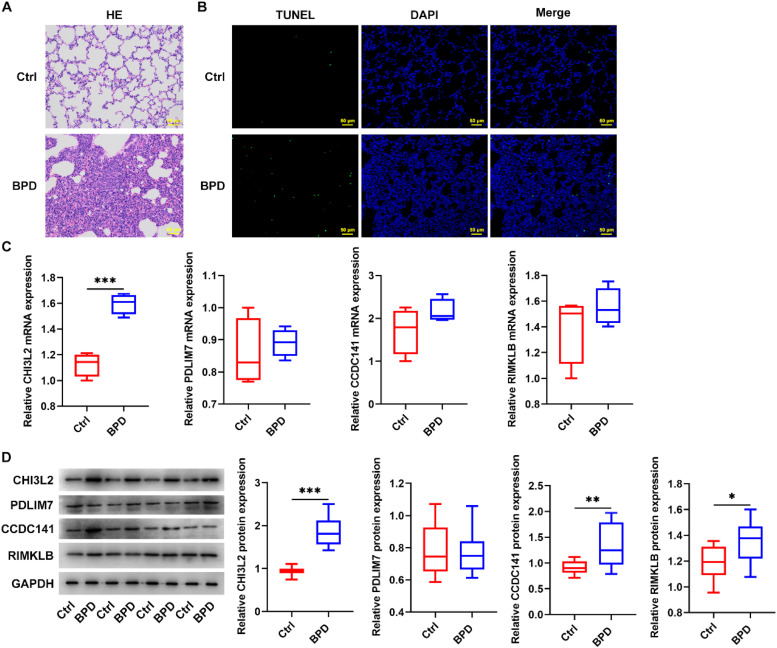
Verification of key marker expression in BPD model mice. (A) Hematoxylin-Eosin (H&E) staining was used to evaluate the pathological changes of lung tissues in the control group and BPD group. (B) Terminal deoxynucleotidyl transferase dUTP Nick-End Labeling (TUNEL) staining was used to analyze the number of TUNEL-positive cells in the lung tissues of newborn mice in the control group and BPD group. (C) Real-Time Polymerase Chain Reaction (RT-qPCR) was used to screen key markers and detect the expression levels of CHI3L2, PDLIM7, CCDC141, and RIMKLB. (D) Western blot was used to screen key markers and detect the expression levels of CHI3L2, PDLIM7, CCDC141, and RIMKLB.

First, lung tissue from newborn BPD mice was obtained. HE-staining was used to detect pathological changes in the lung tissue, and TUNEL staining was performed to observe apoptosis of lung cells. The results showed that, compared with the control group, the lung tissue structure of newborn mice in the BPD group was disordered, the alveolar walls were ruptured and destroyed, alveolar simplification was evident with fewer large fused alveolar structures, the alveolar interstitial edema was widened, and the number of TUNEL-positive cells in the lung tissue increased (Fig. 10A‒B). Then, RT-qPCR and WB were used to screen key markers, and the expression levels of CCDC141, CHI3L2, PDLIM7, and RIMKLB were detected. The results demonstrated that compared to the control group, the expression levels of CCDC141, CHI3L2, and RIMKLB in the lung tissue of newborn mice in the BPD group were upregulated to varying degrees. At the tissue level, PDLIM7 expression showed no statistically significant change (p > 0.05, Fig. 10C‒D) in stark contrast to the consistent upregulation of CHI3L2, CCDC141, and RIMKLB. This lack of PDLIM7 upregulation appears to result from antagonistic immune remodeling. Specifically, lymphoid lineages exhibited marked depletion of T and B cells (T-cells CD4+ naive: p < 0.001; B-cells naive: p = 0.001; Fig. 8A), while myeloid compartments showed significant neutrophil expansion (p = 0.005; Fig. 8A) and an upward trend in the resting NK cell population (p = 0.044; Fig. 8A). This microenvironmental counterbalancing ‒ T/B-cell exhaustion versus neutrophil infiltration ‒ masks PDLIM7′s context-dependent functions.

Critically, these immune alterations align with causal pathology. Mendelian randomization analysis confirmed that neutrophils directly impair lung function (FEV1/FVC reduction, Inverse Variance Weighted Odds Ratio [IVW OR] = 0.966, 95% CI 0.940–0.993, p = 0.015, Fig. 9). Collectively, the experimental immune dysregulation and genetic causality establish that PDLIM7′s bulk-tissue silence reflects biological antagonism that can only be resolved through single-cell analysis. Additionally, we found that CCDC141, CHI3L2, and RIMKLB may play pivotal roles in BPD pathogenesis.

## Discussion

Bronchopulmonary Dysplasia (BPD) is the most common chronic lung disease of prematurity. It has a multifactorial pathogenesis in which inflammation and immune dysregulation play central roles.^15^ As a chronic condition, BPD can exert long-term effects that persist into adulthood. Emerging evidence indicates an association between BPD and an increased risk of developing Chronic Obstructive Pulmonary Disease (COPD). This association significantly impacts the long-term respiratory prognosis of affected children.^6^ Currently, no specific preventive or therapeutic agents are available to effectively alleviate BPD.^16^ A wealth of clinical and experimental evidence underscores the significance of inflammatory processes in the pathogenesis of BPD. These processes are characterized in the lungs of affected infants by infiltration of neutrophils and macrophages, accompanied by elevated levels of various inflammatory cytokines and chemokines.^17^

To unravel the complex pathogenesis of BPD, our study systematically unravels the complex pathogenesis of Bronchopulmonary Dysplasia (BPD) by integrating multi-omics data and Mendelian randomization. We identified a novel four-gene signature and demonstrated a causal relationship between genetically determined neutrophil counts and lung function, thereby providing new insights into the immune mechanisms underlying BPD. The following sections will detail the functional roles of these signature genes, elucidate the pathways they modulate, and explain how our findings contribute to addressing the urgent need for improved diagnostic and therapeutic strategies in the management of BPD.

CHI3L2 is a member of the chitinase-like protein family; however, it lacks chitinase activity due to a mutation in its catalytic center.^18^ CHI3L2 encodes chitinase-like proteins, which are likely involved in tissue remodeling during inflammation and development. Various cell types, such as macrophages, articular chondrocytes, and synovial cells, secrete proteins encoded by the CHI3L2 gene in response to inflammatory conditions.^19^ n human macrophages, CHI3L2 has been observed to be upregulated by IL-4 and TGF-β.^20^ Areshkov et al. suggested that CHI3L2 induces phosphorylation of ERK1/2, similar to NGF, which inhibits cell mitosis and proliferation.^19^ Sanfilippo et al. argued that CHI3L2 represents a promising gene potentially involved in the pivotal processes underlying Alzheimer’s disease.^21^ Furthermore, Comabella et al. proposed that CSF CHI3L2 could serve as a prognostic protein biomarker associated with long-term disability progression in patients with progressive MS.^22^ In the context of BPD, CHI3L2 may influence the occurrence and development of neonatal lung diseases through similar mechanisms. Its mediated inflammatory response and possible neuroimmune interactions may be closely related to the abnormal inflammatory response and disordered tissue repair observed in preterm infants exposed to hyperoxia. Notably, CHI3L2 is indeed expressed in lung tissue, providing a basis for its direct involvement in the pathological processes of BPD.

PDLIM7 functions as a suppressor of p53, diminishing p53’s pro-apoptotic activity and promoting mitosis.^23^ It has been observed that the perturbation profiles of PDLIM7 closely mirror those of MYD88 in regulating pro-inflammatory genes.^24^ In line with this, a double knockdown of PDLIM7 and PDLIM2 in Granulocyte-Macrophage Colony-Stimulating Factor Bone Marrow Cells (GM-CSF-BMCs) led to a significant increase in the production of pro-inflammatory cytokines, namely IL-6, IL-12β, and G-CSF, compared to control cells or those with a single knockdown. This evidence implies that PDLIM7 and PDLIM2 could form heterodimers that collaboratively expedite NF-κB p65 subunit degradation and inhibit inflammatory responses.^25^ Additionally, Matic et al. revealed that the in vitro silencing of PDLIM7 resulted in a downregulation of Smooth Muscle Cell (SMC) markers, disruption of the actin cytoskeleton, decreased cell spreading, and increased proliferation.^26^ They found that the absence of PDLIM7 markedly affects the cell’s ability to rearrange the actin cytoskeleton. Our findings suggest that the overexpression of PDLIM7 in infants with BPD may be an adaptive response. Considering the characteristic alveolar simplification ‒ rather than alveolar obstruction ‒ and abnormal pulmonary vascular development in BPD, PDLIM7 may play a key role in preserving the morphology and facilitating the repair of alveolar epithelial cells and pulmonary microvascular endothelial cells by stabilizing the cytoskeleton and inhibiting excessive inflammation, thereby offering a new perspective for understanding the impaired development of alveolar structure in BPD.

While the direct role of CCDC141 in BPD pathogenesis remains unknown, its significant upregulation in exosomes during influenza A virus infection suggests a potential, yet unexplored, function in the pulmonary antiviral or innate immune response.^27^ This indirect evidence positions CCDC141 as a compelling candidate for future investigation into its specific role within the dysregulated immune microenvironment characteristic of BPD.

Similarly, the potential involvement of RIMKLB in BPD is inferred from its role in modulating immune responses in other contexts. For instance, in oncology, RIMKLB-synthesized NAAG can suppress anti-tumor immunity. By analogy, aberrant RIMKLB activity in the developing lung may disrupt local immune homeostasis.^28^ This hypothesis, while speculative, warrants targeted validation to assess RIMKLB's impact on lung immune responses in the context of neonatal injury.

Alterations in T-cell function associated with BPD correspond with significant changes in thymic structure, including accelerated T-cell maturation and a significant reduction in the number of thymic nurse cells.^29^ This is consistent with our results on immune infiltration. Furthermore, a positive correlation was observed between CCDC141, CHI3L2, and RIMKLB with CD8+ T-cells, resting memory CD4+ T-cells, resting mast cells, and naïve B-cells, whereas a negative correlation was observed with PDLIM7. In contrast, these genes were negatively correlated with neutrophils, activated mast cells, M0-type macrophages, and resting Natural Killer (NK) cells, and positively correlated with PDLIM7.^30^ These findings underscore the complexity of immune cell abnormalities in children with BPD and propose potential therapeutic targets for immunomodulation.

To investigate the molecular mechanisms that might drive the observed immune cell imbalances, we performed Gene Set Enrichment Analysis (GSEA). The results revealed that the signature genes were significantly enriched not only in immune-related pathways but also in specific metabolic processes. These include sulfur metabolism, associated with genes such as CCDC141 To investigate the molecular mechanisms that might drive the observed immune cell imbalances, direct relevance of these metabolic pathways to Bronchopulmonary Dysplasia (BPD) is not yet established, we hypothesize a plausible link through oxidative stress, a well-established driver of BPD pathogenesis, grounded in the fundamental roles of these pathways. Sulfur metabolism is integral to glutathione biosynthesis, a key antioxidant. Its dysregulation could exacerbate oxidative lung injury. Meanwhile, perturbations in aminoacyl-tRNA synthetase activity may reflect disrupted protein homeostasis under cellular stress. Thus, beyond reinforcing the role of immune dysregulation, these metabolic associations offer a novel and testable mechanistic framework ‒ comprising hypotheses and models ‒ focused on oxidative stress and provide a clear direction for experimental validation and clinical studies into BPD pathology.

The biological insights gained from the GSEA and immune infiltration analyses must be considered alongside their methodological limitations. A key limitation pertains to the immune cell infiltration profiles, which were inferred using the CIBERSORT algorithm, on bulk RNA-seq data from limited sample cohorts. While this computational approach provides valuable, hypothesis-generating insights ‒ especially given the challenges in procuring large neonatal clinical samples ‒ the results remain inferential. Therefore, future validation using complementary technical methods, such as flow cytometry or immunohistochemistry on primary patient tissues, is essential. Such validation will conclusively confirm the immune landscape suggested by our in-silico analysis and spatially resolve the proposed cellular dynamics.

To reconcile these findings, we employed a genetic approach to redefine the role of neutrophils. This study is the first to explore the causal relationship between neutrophil count and pulmonary function (FEV1/FVC) using a two-sample Mendelian randomization (MR) framework based on large-scale GWAS data. Conceptually similar to randomized controlled trials, the MR design effectively mitigates confounding biases and reverse causality inherent in observational studies. Our analysis, bolstered by rigorous instrumental variable selection and sensitivity analyses ‒ including MR-Egger regression, which showed no evidence of horizontal pleiotropy ‒ suggests a causal association between genetically predicted higher neutrophil count and lower odds of impaired lung function (Odds Ratio [OR] < 1).

This finding initially appears paradoxical, given the well-established role of neutrophilic inflammation in the pathogenesis of BPD.^31^ However, this apparent contradiction can be reconciled by distinguishing between a genetic predisposition to baseline neutrophil levels and the acute, dysregulated neutrophil activity observed in active disease. Mendelian Randomization (MR) assesses the former ‒ a lifelong, steady-state exposure reflecting baseline neutrophil levels ‒ which may play a distinct, potentially protective role in long-term lung development and homeostasis, for instance, by conferring a robust baseline defense against subclinical infections. This interpretation is further supported by our observation that the key biomarker genes exhibited marked differential expression in peripheral blood at 28-days postnatal age, but not in umbilical cord blood. This finding implicates their role in postnatal events such as hyperoxia and inflammation.

The context-dependent duality of neutrophil function, which can range from tissue-destructive to immune-regulatory, underscores the complexity of their role in Bronchopulmonary Dysplasia (BPD). In homeostatic conditions, neutrophils contribute to immune surveillance, microbial defense, and tissue repair. However, in the pathological microenvironment of BPD ‒ characterized by hyperoxia and barotrauma ‒ this balance is disrupted, leading to a predominant pro-inflammatory, tissue-damaging phenotype. Therefore, our Mendelian Randomization (MR) finding, which highlights a genetically influenced protective role of baseline neutrophil capacity, does not refute the well-established detrimental impact of acute, dysregulated neutrophilic inflammation. Instead, it reveals a previously underappreciated dimension of neutrophil biology in lung development. This novel perspective, which distinguishes between beneficial genetic predisposition and harmful acquired activation, is critical in light of the current inefficacy of treatments aimed at modifying the long-term prognosis of BPD.^6^ Building on this insight, the identified biomarker genes functionally linked to neutrophil-driven immune dysregulation hold significant potential for improving prediction, risk stratification, and the development of targeted therapeutic strategies.

Notwithstanding these insights, our study has limitations. The primary constraint is the sample size derived from public databases, which may introduce selection bias, even though the findings were validated in an external cohort. Furthermore, the mechanistic roles of the identified genes, particularly CCDC141 and RIMKLB, require further investigation through molecular experiments and larger prospective clinical studies. This is necessary to fully substantiate their clinical utility and pathogenic involvement in BPD. Addressing these limitations ‒ particularly through orthogonal validation of immune cell subsets and functional dissection of the candidate genes ‒ will be essential to translate these hypotheses into mechanistic insights and, ultimately, clinical applications.

## Conclusion

Our study identified four potential biomarkers (CCDC141, CHI3L2, RIMKLB and PDLIM7) for BPD. This comprehensive analysis offers novel insights into the immune characteristics and risk factors related to neonatal BPD, thereby enhancing our understanding of the disease's mechanism.

## Authors’ contributions

YS, JY, JD, AC, FL and YC conceived, designed the study, acquired and interpreted the data. JHT and HHW performed the experiments. All authors read and approved the final manuscript.

## Consent for publication

Not applicable.

## Ethics approval and consent to participate

Animal experiments were approved by the Laboratory Animal Ethics Committee of Fudan University. This research involved animal subjects and it complies with ARRIVE guidelines. This study was conducted in full compliance with both the National Standard of China (GB/T 35892-2018) and the international ARRIVE 2.0 guidelines. The animal procedures received prior approval from the Animal Ethics Committee of Fudan University (Approval n° 2023-213), with detailed documentation available in the Supplementary Materials.

## Data availability

The gene expression datasets (GSE32472 and GSE108754) reanalyzed in this study are publicly available from the NCBI Gene Expression Omnibus (GEO) repository at the following URLs: GSE32472: https://www.ncbi.nlm.nih.gov/geo/query/acc.cgi?acc=GSE32472; GSE108754: https://www.ncbi.nlm.nih.gov/geo/query/acc.cgi?acc=GSE108754. Further details on data sources and processing are provided in the Materials and Methods section.

The datasets used and/or analyzed during the current study are available from the corresponding author upon reasonable request.

## Conflicts of interest

The authors declare no conflicts of interest.

## Appendix Supplementary materials

Image, application 1

Image, application 2

Image, application 3

Image, application 4

Image, application 5
