# Supplementary figures and images for: Multi-omics integration and machine learning identify a novel gene signature consisting of CCDC141, CHI3L2, RIMKLB, and PDLIM7 in bronchopulmonary dysplasia associated with neutrophil-driven immune dysregulation
Source: Clinics (Sao Paulo). 2026 Jul 14;81:101059. doi: 10.1016/j.clinsp.2026.101059 (PMC13382404; doi:10.1016/j.clinsp.2026.101059)

# Cross-Validation Performance Metrics

Valid folds: 5/5

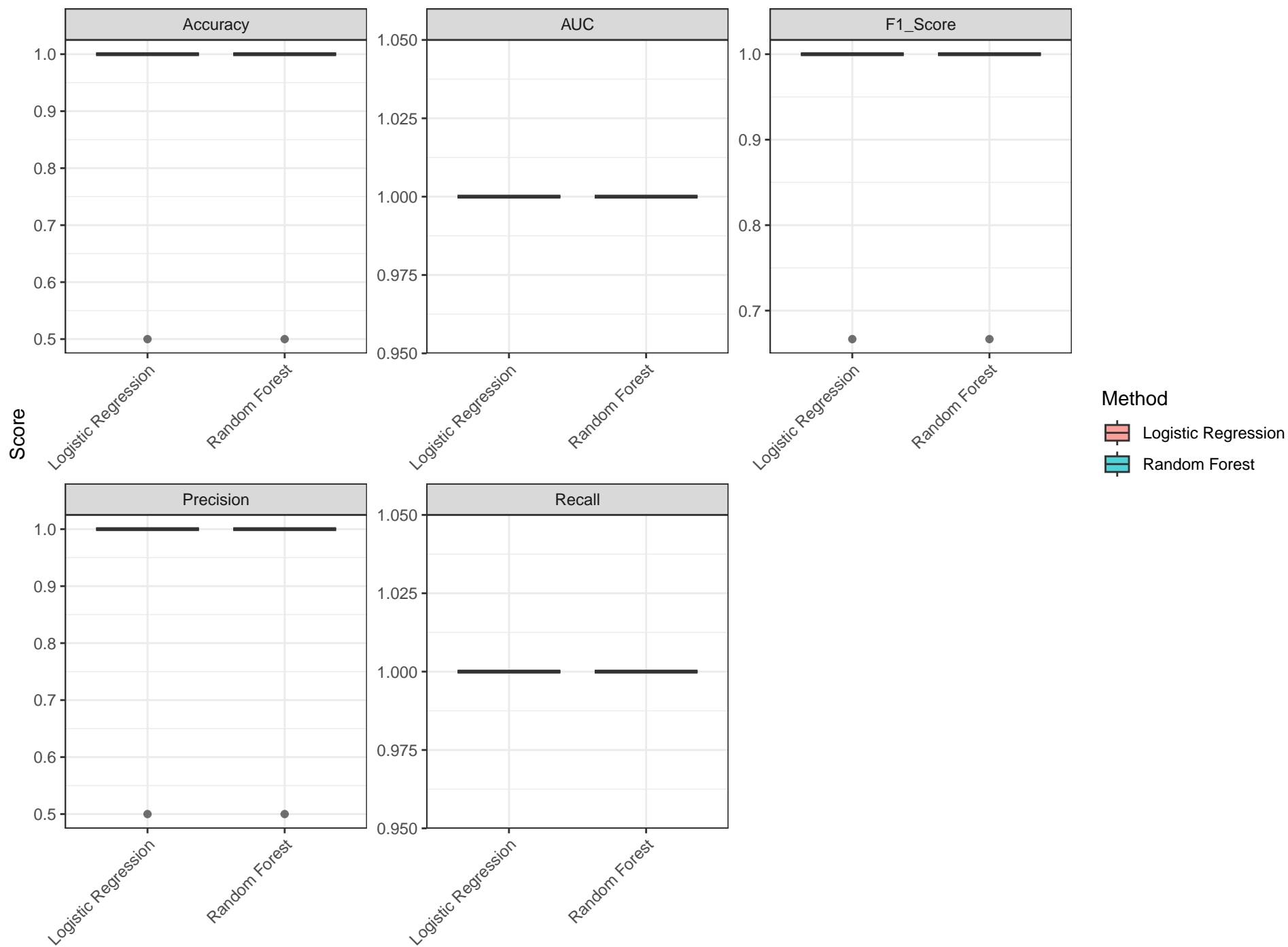

Supplement: Supplementary file 4 [file mmc4.zip › 1.Supplementary_Figure_CV_Performance.pdf]

# Permutation Test Results

Dashed lines show real model AUC

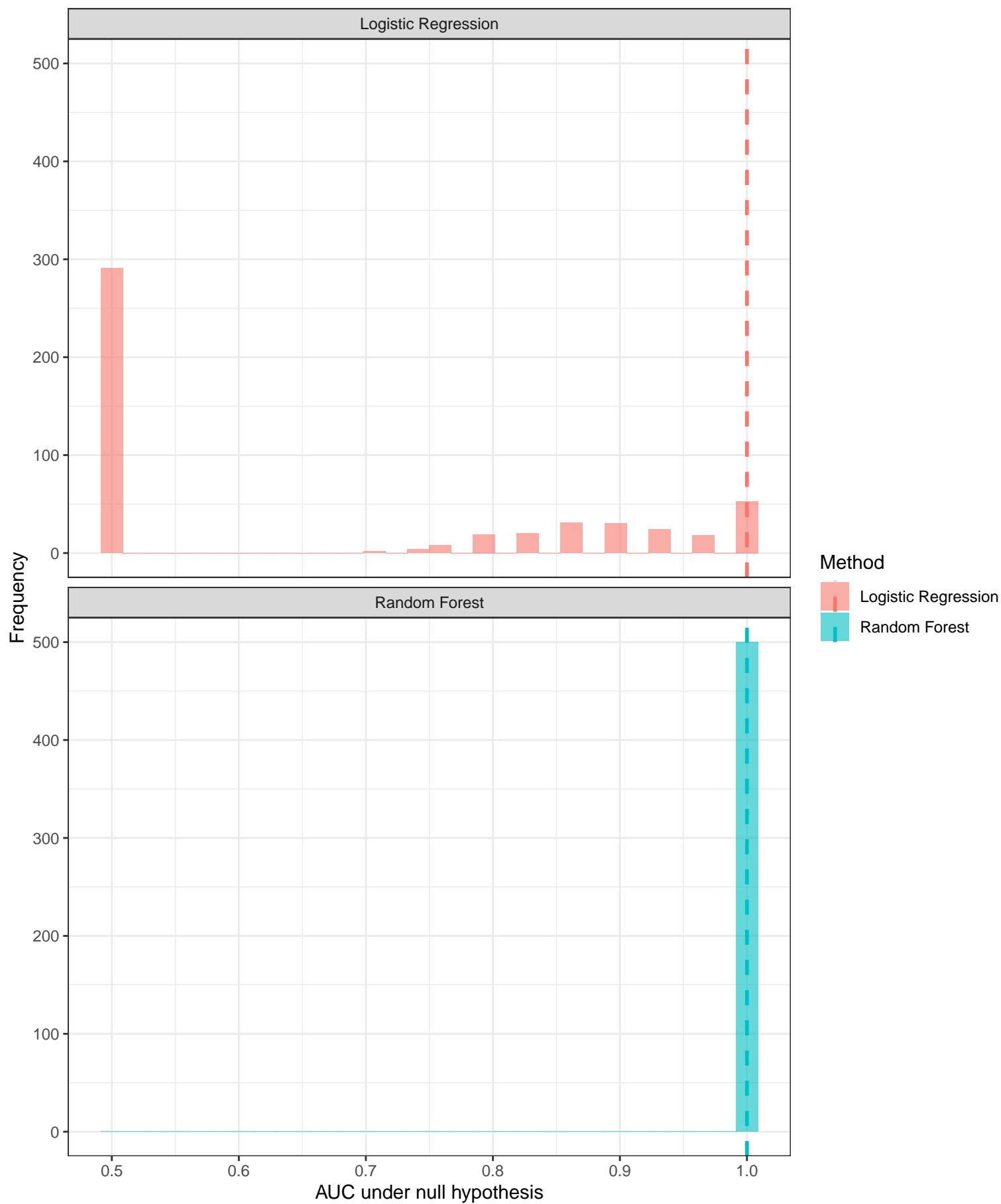

Supplement: Supplementary file 5 [file mmc5.zip › 2.Supplementary_Figure_Permutation_Test.pdf]
